# Intermolecular Potential Energy Surfaces and Bound State Calculations of Rg–CuF (Rg = Ar, Kr, Xe): Insights into the Nature of Noble Gas–Metal Bonding

**DOI:** 10.3390/molecules31173025

**Published:** 2026-08-28

**Authors:** Xiang Li, Zhuang Liu, Kangning Peng, Wei Luo, Rui Zheng

**Affiliations:** 1 College of Physics and Electronic Information, Nanchang Normal University, Nanchang 330032, China; lixiang1992@ncnu.edu.cn (X.L.); zhuangliu@ustc.edu.cn (Z.L.); 2 College of Physical Education, Nanchang Normal University, Nanchang 330032, China; pengkangning1995@163.com; 3 School of Science, East China University of Technology, Nanchang 330100, China; luowei@ecut.edu.cn

**Keywords:** noble gas–metal complexes, potential energy surfaces, spectroscopic parameters, isotopic effects

## Abstract

High-precision two-dimensional intermolecular potential energy surfaces (PESs) for Rg–CuF (Rg = Ar, Kr, Xe) were constructed at the coupled-cluster singles and doubles with non-iterative triples [CCSD(T)] level by employing aug-cc-pVXZ (X = D, T, Q) basis sets, and the energies were extrapolated to the complete basis set (CBS) limit. All three complexes exhibit a consistent topological pattern: the global minimum corresponds to a collinear Rg–Cu–F configuration, and the local minimum corresponds to an anti-linear Rg–F–Cu configuration. As the atomic number of noble gas increases, the Rg–Cu equilibrium distance lengthens while the binding strength remarkably enhances. Bound state calculations were performed based on these PESs to yield rotational levels, which can be used to derive the intermolecular vibrational frequencies, molecular structures and spectroscopic parameters for all primary isotopologues. The predicted rotational constants *B* are in excellent agreement with the experimental observations, attaining a sub-MHz accuracy at the AVTZ level for Kr–CuF and at the CBS limit for Ar–CuF and Xe–CuF. Vibrational wavefunction analysis reveals that the intermolecular vibrational modes of Kr–CuF and Xe–CuF are highly localized, consistent with the pronounced molecular rigidity observed experimentally. Isotopic effect analysis reveals a well-defined linear relationship between the changes in the rotational constant *B* and the intermolecular vibrational frequency in relation to the reduced mass of the complex, which provides a reliable basis for predicting spectroscopic parameters of unobserved isotopologues. Symmetry-adapted perturbation theory (SAPT) energy decomposition further demonstrates that the Rg–Cu interaction is dominated by induction forces, with significant contributions from dispersion and electrostatics, and exhibits notable charge transfer character. This polarization and orbital overlap transcend the conventional van der Waals picture and reveal a partially covalent nature in noble gas transition metal interactions.

## 1. Introduction

Noble gases have long been regarded as chemically inert owing to their closed-shell electronic configurations. However, since the successful synthesis of compounds such as HArF [1] and [AuXe_4_]^2+^ [2], the bonding mechanism between noble gases and transition metals has attracted sustained research interest. Among these systems, noble gas–transition metal halide complexes (Rg–MX, Rg = Ar, Kr, Xe; M = Cu, Ag, Au; and X = F, Cl, Br, I) possess well-defined linear triatomic structures and information-rich rotational spectra, making them ideal model systems for probing such interactions. Unlike typical van der Waals (vdW) complexes, Rg–MX systems exhibit unusually short bond lengths and substantial binding energies, placing their bonding character in an intermediate regime between pure vdW interactions and covalent bonds [3,4,5,6,7,8,9,10,11,12]. They therefore provide a valuable opportunity to trace the continuous transition from non-covalent interactions to chemical bonding.

Experimentally, Gerry and co-workers investigated the pure rotational spectra of a series of Rg–MX complexes using laser ablation coupled with pulsed jet cavity Fourier transform microwave (FTMW) spectroscopy. Their studies initially focused on the argon-containing species Ar–AgX (X = F, Cl, and Br) [3] and Ar–CuX (X = F, Cl, and Br) [4]. These measurements established that the complexes adopt linear Rg–M–X equilibrium geometries. This structural motif was subsequently identified for the corresponding krypton and xenon complexes, including Kr–AgCl [5], Kr–AgF [6], Xe–AgF, and Xe–AgCl [7] for silver halides; Kr–CuF, Kr–CuCl [8], Xe–CuF, and Xe–CuCl [9] for copper halides; and Ar–AuCl, Kr–AuCl [10], Ar–AuF, Ar–AuBr [11], and Xe–AuF [12] for gold halides.

Across this series, the Rg–M bond lengths are substantially shorter than the sums of the corresponding atomic van der Waals radii. Moreover, the binding energies increase markedly from Ar to Xe, ranging from approximately 14 to 23 kJ mol^−1^ for the Ar–AgX complexes [3,5] to approximately 100 kJ mol^−1^ for Xe–AuF [12]. These values are considerably larger than the approximately 10 kJ mol^−1^ typically associated with conventional van der Waals interactions [11]. In addition, anomalous changes in the nuclear quadrupole coupling constants provide compelling evidence for substantial charge redistribution upon complex formation. For example, the ^83^Kr nuclear quadrupole coupling constant of Kr–CuF is 128.8 MHz, in marked contrast to the zero value for the isolated Kr atom [8]. Similarly, large ^131^Xe and ^197^Au nuclear quadrupole coupling constants have been reported for Xe–AuF [12]. These observations indicate pronounced electron density redistribution accompanying Rg–M bond formation.

Theoretical investigations have improved the understanding of bonding in these systems. Belpassi et al. [13] precisely quantified charge transfer from Rg to M through charge-displacement analysis. Ghanty [14] and Mou [15] investigated the geometries and binding energies of these complexes at the MP2 and CCSD(T)/CBS levels, respectively. Evans et al. [16] reported precise binding energies of Ar–CuF and Ar–CuCl at the complete basis set (CBS) limit, and Pan et al. [17] classified this interaction as a novel type of weak interaction. Despite the increasingly refined understanding at the electronic structure level, accurately reproducing and predicting experimentally observable spectroscopic parameters from first principles still critically depends on the construction of high-precision intermolecular potential energy surfaces (PESs) and rigorous bound-state calculations.

In recent years, our group has made significant progress in PES construction and spectroscopic prediction for Rg–MX systems. Previously, high-precision PESs have been constructed for Ar–AgX [18], Ar–CuX [19], and Kr–AgX [20,21], successfully predicting pure rotational transition frequencies and spectroscopic parameters. These studies also demonstrated that the impact of basis set superposition error (BSSE) correction on PES quality varies across systems, which serves as a reminder that computational strategies must be evaluated according to their inherent characteristics.

However, systematic PES studies encompassing the full Rg–CuF (Rg = Ar, Kr, Xe) series remain absent, and several crucial questions remain to be resolved. Firstly, the convergence behavior and error cancellation patterns across the entire series under different basis sets have not been characterized. Secondly, it is still unclear how the mass of the heavier noble gas atom and the depth of the potential well jointly determine the localization of intermolecular vibrational wavefunctions and the ordering of energy levels. Moreover, the quantitative influence of natural isotopic substitution on rotational constants and vibrational frequencies requires a comprehensive study based on high-precision PESs.

In this work, we constructed high-precision two-dimensional PESs for Rg–CuF (Rg = Ar, Kr, Xe) at the CCSD(T)/aug-cc-pVXZ (X = D, T, Q) level and at the complete basis set (CBS) limit. Based on the optimal PESs obtained, rigorous bound-state calculations were performed to characterize the intermolecular vibrational wavefunctions and to accurately predict their spectroscopic parameters, molecular structures, and isotopic effects, and an extensive comparison was made against the available experimental data.

## 2. Results and Discussion

### 2.1. PES Topology and Basis Set Convergence

**Figure 1:**
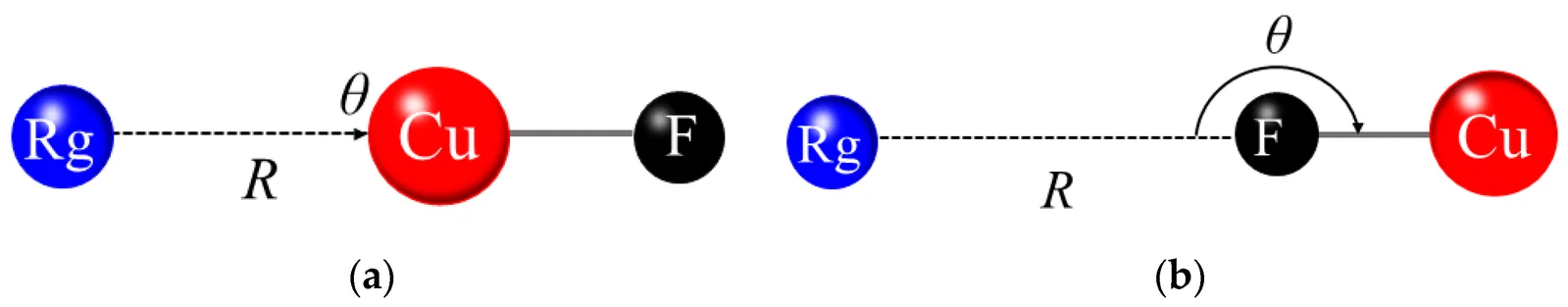
Molecular structure of Rg–CuF. (**a**) the linear configuration; (**b**) the anti-linear configuration.

This work presents a comprehensive computational investigation of the Rg–CuF (Rg = Ar, Kr, Xe) complexes at the CCSD(T) level, employing three Dunning-type basis sets (aug-cc-pVDZ, aug-cc-pVTZ, and aug-cc-pVQZ, hereafter denoted as AVDZ, AVTZ, and AVQZ) alongside two complete basis set (CBS) extrapolation schemes (CBS-dz+tz and CBS-tz+qz). The geometric parameters on the PESs are summarized in Table 1 as well as the corresponding interaction energies. Each set of three values in parentheses in the table represents the intermolecular distance (*R*), the angle (*θ*), and the binding energy, respectively. The specific definitions of *R* and *θ* can be found in Section 3.1. As shown in Table 1, all three Rg–CuF complexes exhibit a consistent topological pattern on their PESs: the global minimum corresponds to a linear Rg–Cu–F configuration (*θ* = 0°), the local minimum is an anti-linear Rg–F–Cu configuration (*θ* = 180°), and a saddle point connects these two minima. Figure 1a and Figure 1b illustrate the schematic diagrams of the linear and anti-linear structures, respectively. At the global minimum, the equilibrium Rg–Cu bond length at the CBS-tz+qz limit increases from 2.615 Å for Ar to 2.713 Å for Kr, and further to 2.838 Å for Xe, consistent with the increasing atomic radii of the noble gases. Concurrently, the binding energy deepens significantly across the series, ranging from –3817.23 cm^−1^ for Ar–CuF to –6086.10 cm^−1^ for Xe–CuF. This substantial strengthening correlates with the higher polarizability of the heavier noble gas atoms and confirms that induction and dispersion forces are the primary drivers stabilizing the Rg–Cu interaction.

In contrast, the potential energies of the anti-linear local minima range from −192.25 to −349.20 cm^−1^, accounting for merely 5–6% of those of the corresponding global minima. Furthermore, its enhancement with increasing Rg atomic size is far less pronounced than that observed in the linear configuration. In this anti-linear geometry, the Rg atom is in direct contact with the highly electronegative F atom. The compact electron cloud of the F atom exhibits low polarizability, resulting in dispersion and induction forces between Rg and F that are substantially weaker than the Rg–Cu interactions. This characteristic aligns with the Ar–AgF system [18], where the binding energy of the anti-linear isomer is approximately 10% of its global minimum. Additionally, the saddle point data elucidate the isomerization pathway between the anti-linear and linear configurations. From Ar to Xe, the saddle point angle *θ* gradually shifts toward the linear geometry (from 140° to 143°), which reflects stronger angular anisotropy in heavier noble-gas systems. More importantly, the isomerization barrier (the energy difference between the saddle points and the global minimum) increases significantly with the mass of the Rg atom. Based on the CBS-tz+qz calculations, the barrier heights for Ar–CuF, Kr–CuF, and Xe–CuF are approximately 3624 cm^−1^, 4574 cm^−1^, and 5749 cm^−1^, relative to its global minimum, respectively. This trend mirrors the behavior reported for the Kr–AgX (X = F, Cl, Br, I) series [20]. Such formidable energy barriers imply that once the system is trapped in the linear global minimum, undergoing isomerization is highly improbable under typical experimental conditions. This further establishes the absolute dominance of the linear Rg–CuF configuration in experimental observations from both kinetic and thermodynamic perspectives.

**Figure 2:**
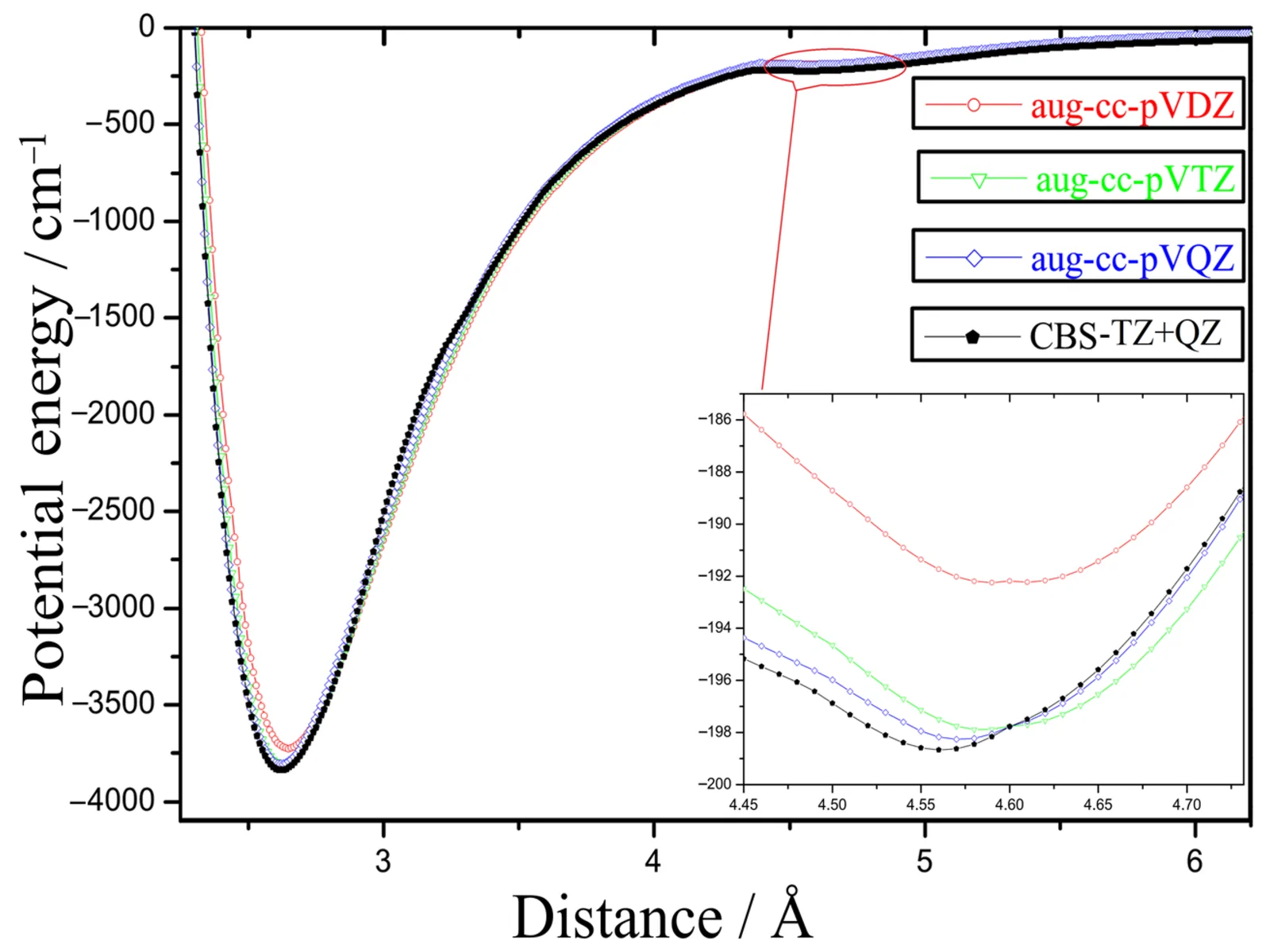
Plot of the potential energy versus intermolecular distance for Ar–CuF in different basis sets.

**Figure 3:**
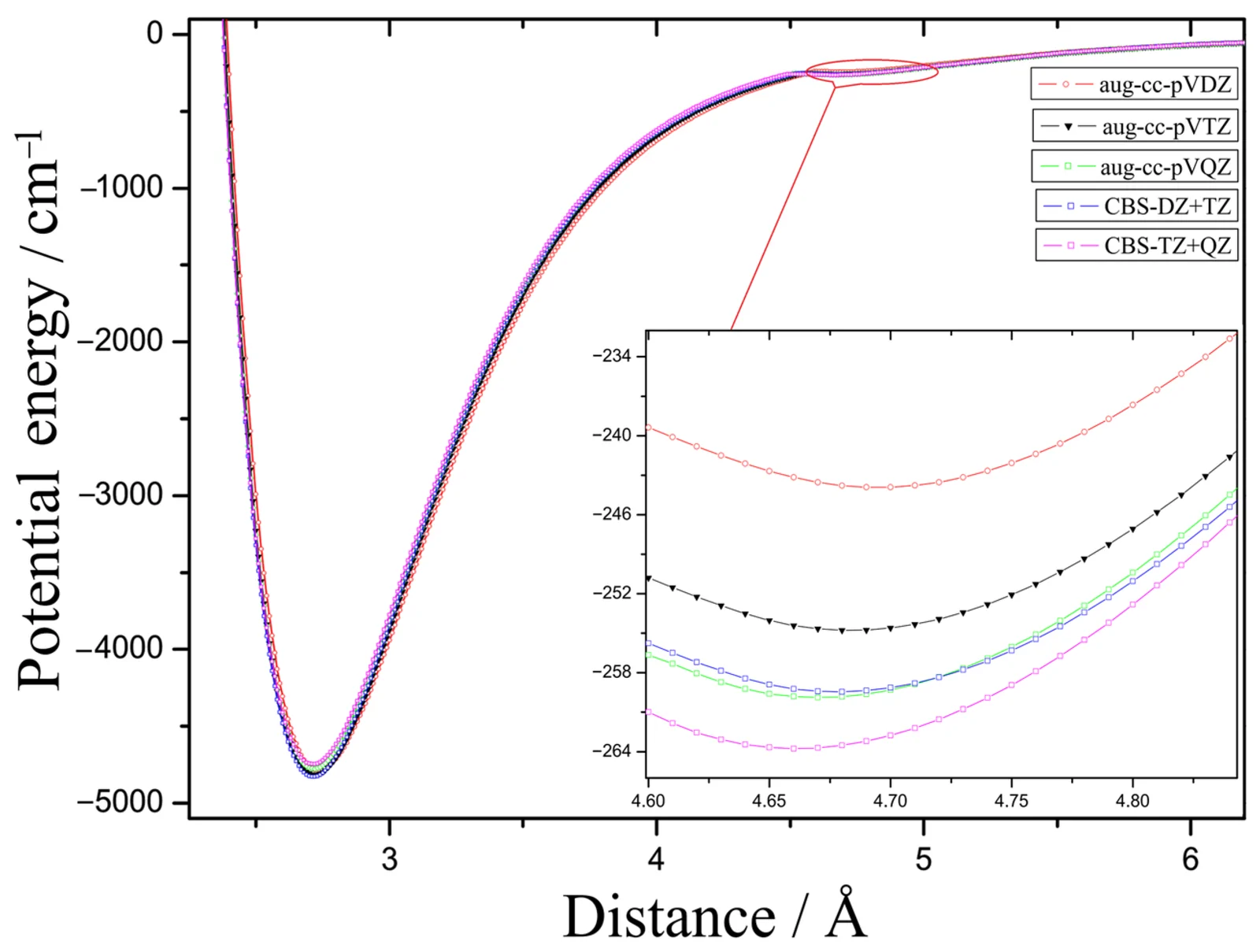
Plot of the potential energy versus intermolecular distance for Kr–CuF in different basis sets.

**Figure 4:**
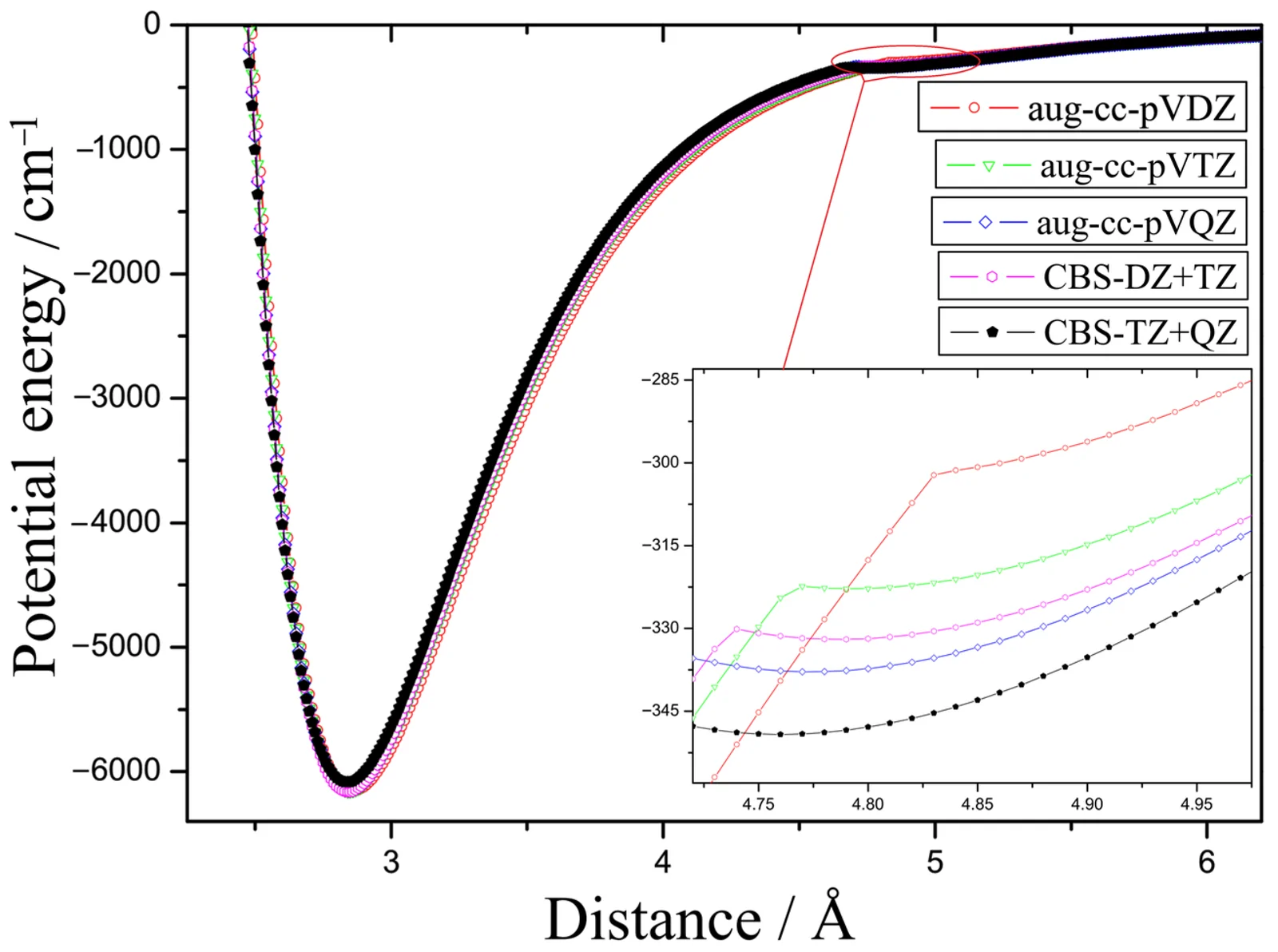
Plot of the potential energy versus intermolecular distance for Xe–CuF in different basis sets.

Figure 2, Figure 3 and Figure 4 plot the potential energy as a function of the intermolecular Rg–Cu distance to illustrate the convergence behavior across basis sets, with magnified insets resolving the local-minimum regions. Along with their magnified insets, clearly illustrate the pronounced sensitivity of the calculated results to the size of basis set. Overall, as the basis set is expanded from AVDZ to the CBS-tz+qz limit, the equilibrium distances at both minima exhibit a systematic contraction. This contraction of bond length is a hallmark of improved electron correlation effects: the inclusion of more comprehensive polarization and diffuse functions allows for a more accurate description of the dispersion forces, thereby deepening the potential well and shifting it toward shorter intermolecular distances.

However, the basis set convergence pathways at the local minima vary significantly among the various systems, as revealed by the insets. For the lighter Ar–CuF complex (inset of Figure 2), the binding energy converges smoothly from AVDZ to the CBS limit. The AVTZ basis set already captures 99.6% of the CBS limit energy, relatively modest basis set requirements for this system. Conversely, for the heavier Kr–CuF and Xe–CuF complexes (insets of Figure 2 and Figure 3), the situation is different. In the Xe–CuF system, the binding energy decreases sharply from the AVDZ level (−301.18 cm^−1^) to the AVQZ (−337.84 cm^−1^) and CBS-tz+qz (−349.20 cm^−1^) limits. The AVDZ basis set captures only about 86%, of the true binding energy and the CBS-dz+tz extrapolated value (−331.97 cm^−1^), derived from AVDZ and AVTZ, is even shallower than that obtained from the single AVQZ basis set. This anomalous behavior highlights a crucial methodological caveat: when describing the diffuse, long-range interactions of heavy noble-gas atoms, double-zeta quality basis sets (AVDZ) exhibit significant deficiencies. Incorporating them into the CBS extrapolation scheme fails to improve accuracy and instead introduces systematic errors.

By synthesizing the analysis of the global and local potential energy curves, the basis set convergence behavior leads to a conclusion of broad methodological significance. When quantitatively describing the local minima of Rg–MX complexes, relying solely on AVDZ or AVTZ level basis sets can lead to substantial deviations. Not only is the Rg–Cu distance systematically overestimated, but the binding energy is also significantly underestimated (up to 14% in Xe–containing systems at the AVDZ level). Therefore, if the research target is to rigorously evaluate the kinetic stability or experimental observability of the anti-linear isomers, employing AVQZ or higher-level basis sets, or performing high-level CBS extrapolations, is indispensable. Meanwhile, the AVTZ basis set already provides a nearly converged description for the Ar–CuF local minimum (with an error of only ~0.5%), offering a valuable reference for balancing computational cost and accuracy. These findings corroborate the systematic evaluation of basis set performances in the Kr–AgX series reported by Luo et al. [20] and together they highlight the need to select the computational level based on the specific physical quantities and target systems under study.

### 2.2. Accuracy Tests for the PESs

The reliability of the constructed PESs for the Rg–CuF (Rg = Ar, Kr, Xe) complexes was evaluated by comparing the theoretically predicted rotational constants with available the experimental data. For the linear Rg–CuF systems, the vibrationally averaged ground state rotational constant (*B*) serves as a sensitive probe for assessing the accuracy of both the underlying PESs and the bound-state calculations. The rotational levels of the three complexes, computed employing various theoretical models, are provided in Table S1 of the Supplementary Material. Subsequently, rotational transition frequencies were obtained directly from the energy levels via bound state calculation. These ab initio transition frequencies were then fitted using SPFIT [22], thereby yielding effective rotational constants *B*. It should be emphasized that *B* was not used as an input for the bound-state calculations or for generating the transition frequencies. It was derived by parameterizing the calculated energy levels to enable a direct comparison with the experimentally fitted spectroscopic constants. The calculated and experimental *B* values for three representative isotopologues, Rg–^63^Cu^19^F (Rg = ^40^Ar, ^82^Kr, ^132^Xe) are summarized in Table 2. Notably, the CBS limit for Ar–CuF was obtained using a three-point extrapolation formula (Equation (3)), whereas a two-point extrapolation formula (Equation (4)) was employed for Kr–CuF and Xe–CuF.

Overall, as the size of the basis set increases from AVDZ to the CBS limit, the calculated rotational constants exhibit a systematic monotonic increase. At the AVDZ level, the calculated *B* values for all three complexes are severely underestimated, directly reflecting the limitations of the AVDZ basis set in capturing long-range dispersion interactions. As polarization and diffuse functions are gradually added (AVTZ → AVQZ), electron correlation effects are more adequately described. Consequently, the intermolecular potential well deepens and shifts toward shorter distances, causing the calculated *B* values to rapidly approach the experimental counterparts. For ^40^Ar–CuF and ^132^Xe–CuF, the highest-level CBS-tz+qz extrapolation demonstrates exceptional predictive capability, yielding absolute deviations of merely 1.032 MHz and 0.881 MHz, respectively. In contrast, ^82^Kr–CuF presents an intriguing phenomenon: the AVTZ basis set yields a nearly perfect prediction (relative error 0.003%), whereas upgrading to AVQZ or two CBS limits widens the deviation, increasing it to –3.858, –5.732, and –6.718 MHz, respectively. This implies a fortuitous error cancellation at the AVTZ level. Specifically, the inaccuracies in describing the Kr core-electron correlation [23], the BSSE correction [24], and scalar relativistic effects happen to perfectly compensate for one another. Although the higher-order AVQZ basis set and CBS limits provide a more rigorous treatment of individual physical effects, they disrupt this delicate balance, resulting in a slight over-correction. Similar phenomena have been documented in the literature; for instance, in the Ar–AgX [18] and Kr–AgX [20] systems, the uncorrected potential energy surfaces or specific basis sets often fortuitously yield more accurate spectroscopic constants. This cross-system evidence suggests that for heavy noble-gas containing Rg–MX complexes, although BSSE is a genuine physical effect [25], applying the CP correction under incomplete basis set conditions may alter the topology of the potential energy surface. This can introduce new systematic errors at certain basis set levels, necessitating a case-by-case evaluation of its applicability [18,20].

**Figure 5:**
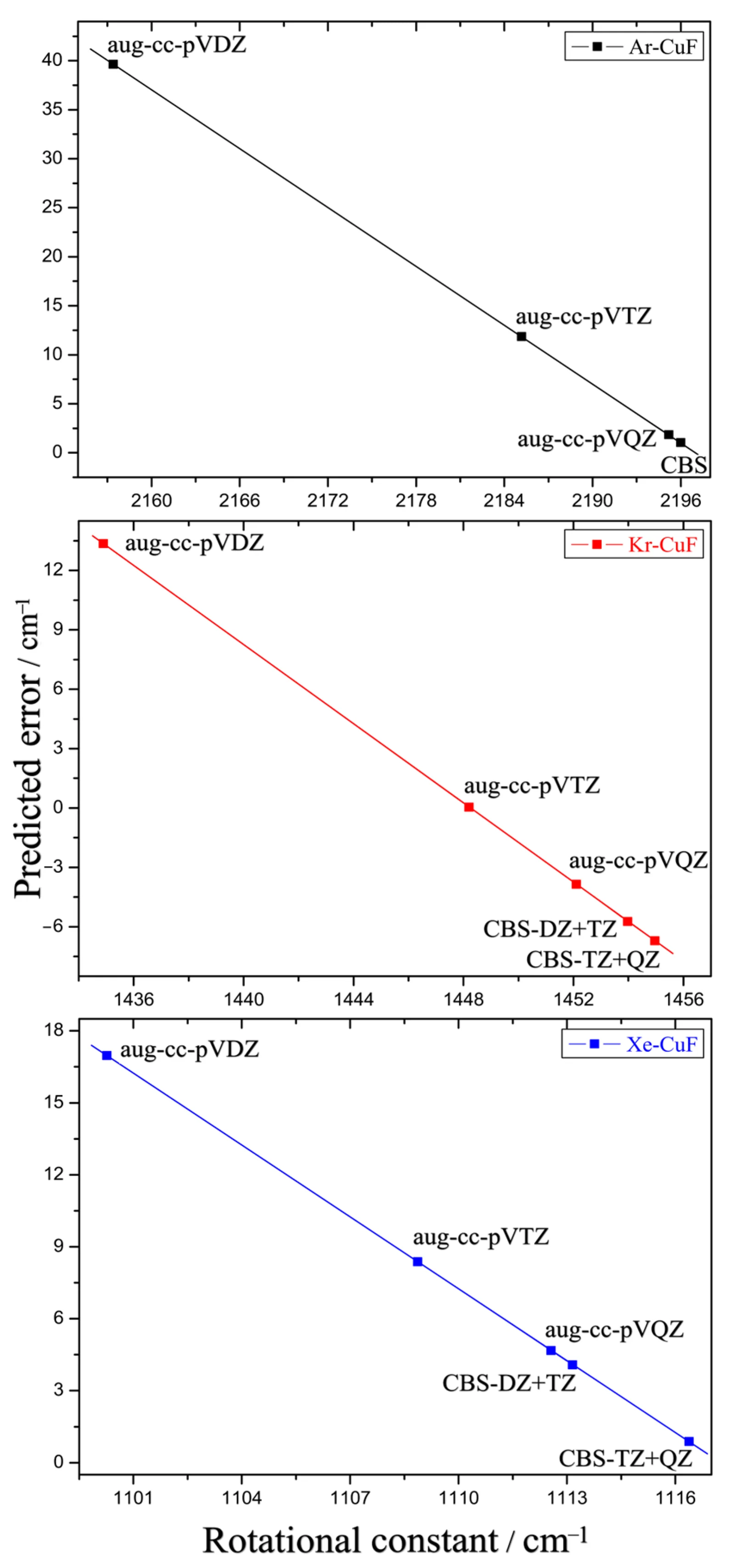
Plot of the prediction errors in the rotational constant *B* for the Rg–CuF (Rg = Ar, Kr, Xe) complexes across different basis sets.

Furthermore, Figure 5 visually reveals the intrinsic correlation between the prediction deviation Δ*B* and the calculated rotational constant *B*. Across all three complexes, as the basis set expands from AVDZ to the CBS limit, an exceptionally perfect linear correlation is observed between Δ*B* and the calculated *B* values. This rigorous linear scaling behavior indicates that the basis set truncation error, when modifying the curvature and the minimum position of the potential energy surface, adheres to a systematic and predictable physical pattern. This linear trend not only further validates the aforementioned conclusion regarding the systematic bond length contraction induced by basis set enlargement, but also provides a potentially effective strategy for predicting higher-accuracy spectroscopic parameters via empirical linear extrapolation at a lower computational cost in future studies.

### 2.3. Intermolecular Vibrational Modes

**Figure 6:**
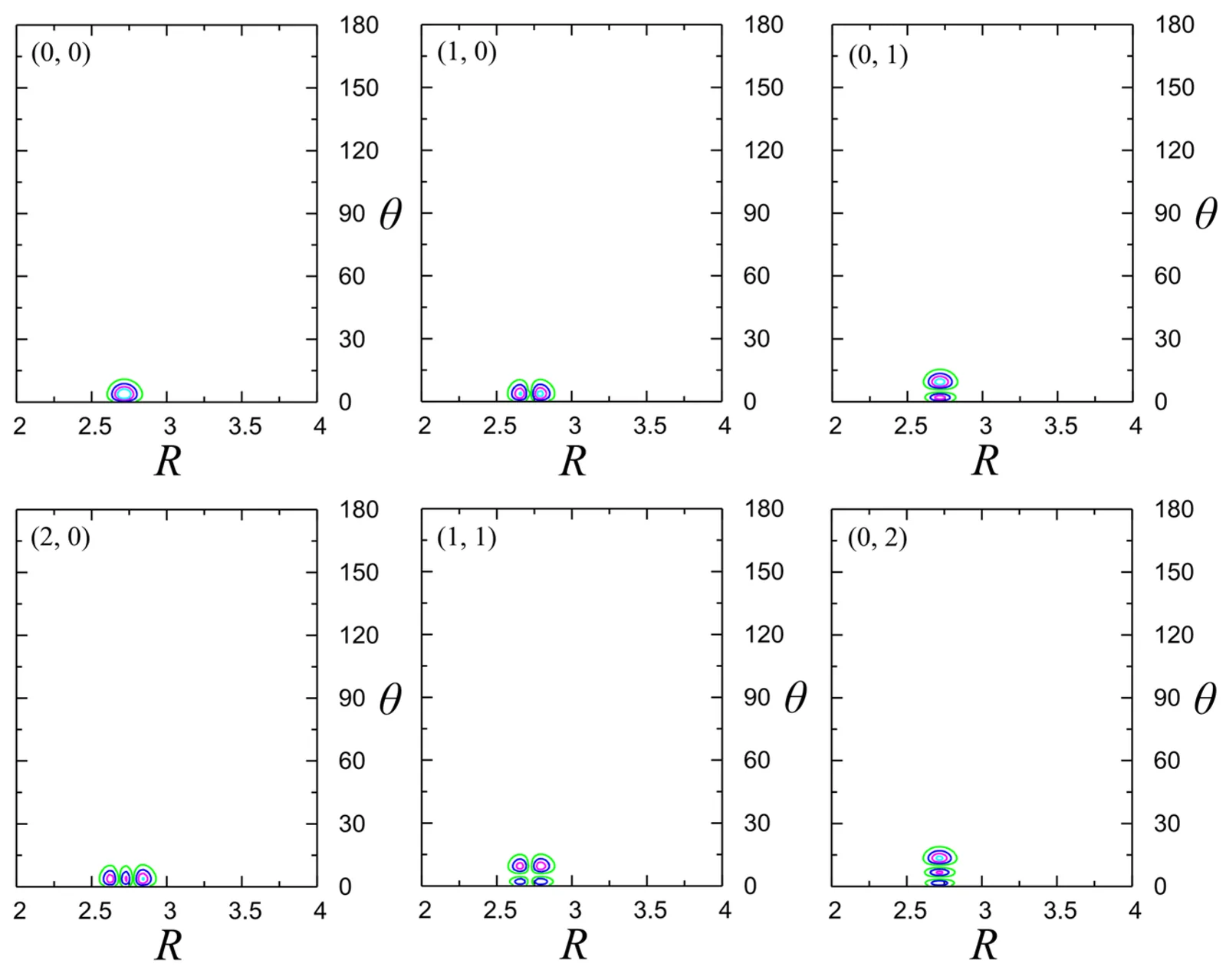
The *R*–*θ* contour plots for the first six low-lying intermolecular vibrational wavefunctions in Kr–CuF.

**Figure 7:**
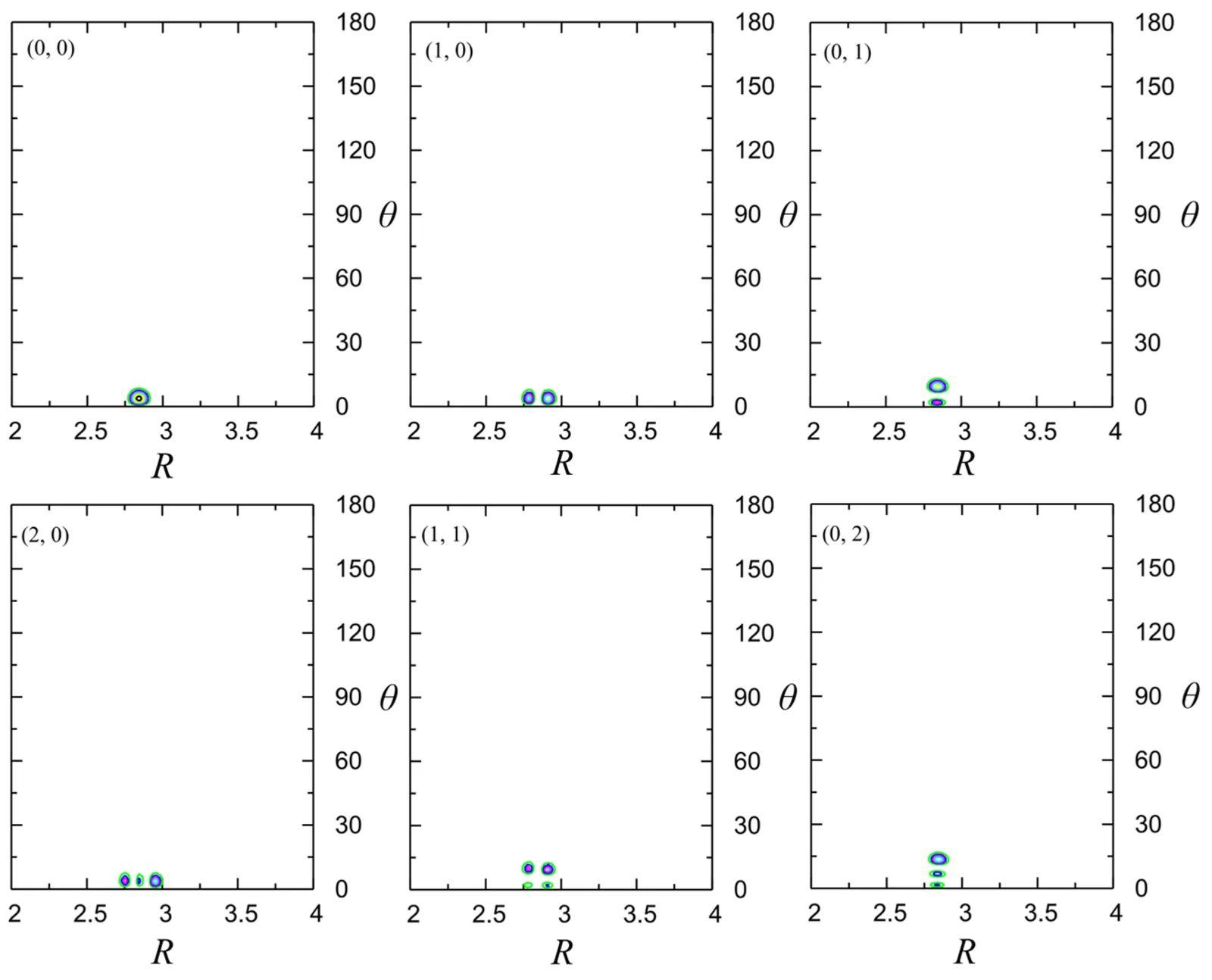
The *R*–*θ* contour plots for the first six low-lying intermolecular vibrational wavefunctions in Xe–CuF.

As discussed above, the CBS-tz+qz limit yields the most accurate results for Ar–CuF and Xe–CuF, while the AVTZ basis set provides the highest accuracy for Kr–CuF. Therefore, all subsequent calculations are based on the respective optimal potential energy surfaces determined for these three complexes. To systematically elucidate the quantum dynamical behaviors of these heavy noble-gas coordination systems and their atom-dependent trends, bound state calculations were conducted on their optimal PESs to determine the intermolecular vibrational states of the Rg–CuF (Rg = Kr, Xe) complexes. The calculated isotopic energy levels and corresponding frequencies are tabulated in Table S2 of the Supplementary Material. The *R*–*θ* contour plots of the wavefunctions for the first six low-lying intermolecular vibrational states are depicted in Figure 6 and Figure 7, with each state assigned by its stretching (*n_s_*) and bending (*n_b_*) quantum numbers.

As depicted in Figure 6 and Figure 7, the topological features of the ground-state wavefunctions clearly demonstrate the rigid linear configurations of both complexes. An inspection of their (0, 0) ground vibrational states reveals that the probability densities are highly localized around the equilibrium angle of *θ* = 0°, strictly confined within a narrow angular region (*θ* < 15°). Along the radial coordinate, the density maxima accurately match the equilibrium distances of the respective systems (approximately 2.7 Å for Kr–CuF and 2.85 Å for Xe–CuF), which is consistent with the larger atomic radii of Xe. Furthermore, owing to the larger reduced mass of the Xe-containing system, its spatial wavefunction distribution is noticeably more compact than that of Kr–CuF, indicating a stronger suppression of zero-point vibrational delocalization. Ultimately, this strong spatial localization provides compelling dynamical evidence that both complexes behave as highly rigid linear molecules in their ground states.

The high regularity of the excited states corroborates the steep topography of the PESs. As the vibrational energy increases, the wavefunctions of both systems exhibit a clear and regular evolution of nodal lines, providing a solid foundation for the definitive assignment of quantum numbers. For the pure stretching vibrational states, namely (1, 0) and (2, 0), the wavefunctions display one and two nodal lines along the *R* coordinate, respectively. Conversely, the nodal lines for the pure bending states, namely (0, 1) and (0, 2), are distributed entirely along the *θ* coordinate. Notably, even in a relatively high bending excited state such as (0, 2), the wavefunctions of Kr–CuF and Xe–CuF remain tightly confined within a narrow angular interval of 30°. This strong spatial confinement clearly demonstrates that the bending PESs for both systems are exceptionally steep, exerting strong restoring forces on the complexes upon bending deformation.

Crucially, the combination band states reveal extremely weak anharmonic coupling and excellent mode decoupling. An analysis of the wavefunctions for the (1, 1) stretch-bend combination states in both complexes shows that their contour plots exhibit a highly regular, grid-like pattern of intersecting nodal lines. The nodes along the *R* and *θ* directions are strictly orthogonal, devoid of any observable topological distortion. This perfect nodal orthogonality is significant from a quantum dynamics perspective, as it indicates that the anharmonic coupling between the intermolecular stretching and bending modes in is virtually negligible. Consequently, despite the progressive enhancement of dispersion interactions from Kr to Xe, the intermolecular dynamical behaviors of these systems in the low-energy regime can be accurately described by a model of two completely decoupled, independent harmonic oscillators.

### 2.4. Spectroscopic Parameters

The rotational levels of the Rg–CuF (Rg = Kr, Xe) complexes, obtained from bound-state calculations based on their respective optimal PESs, are summarized in Table S3 of the Supplementary Material. Accordingly, the corresponding microwave transition frequencies were derived and are tabulated in Tables S4–S6 of the Supplementary Material. Furthermore, the microwave transitions for all isotopologues presented in Tables S4–S6 were fitted using the following Hamiltonian [26]:

(1) $${\hat{H}}_{rot}=B{\hat{J}}^{2}+D{\hat{J}}^{4}$$

This fitting procedure yielded the rotational constant (*B*) and the quartic centrifugal distortion constant (*D*) of ground state for each species. Table 3 presents a detailed comparison of the calculated *B* and *D* with FTMW experimental measurements. The results demonstrate that the selected theoretical strategy yields exceptionally high consistency across the entire isotopologue family. For all isotopologues with available experimental data, the relative errors between the calculated and experimental *B* values are well controlled, with the vast majority of deviations being less than 0.1%. More importantly, the theoretical calculations accurately capture the systematic isotopic shifts in the rotational constants induced by mass variations. Taking ^82^Kr–CuF as example, upon substitution of ^63^Cu with the heavier ^65^Cu, the experimental *B* value decreases from 1448.2467 MHz to 1440.7921 MHz. Our theoretical calculations precisely reproduce this ~7.45 MHz shift. This excellent reproduction of the mass dependence strongly corroborates the direct modulation of the rotational energy levels by the increased reduced mass in the molecular moment of inertia.

The centrifugal distortion constant (*D*) reflects the degree of bond stretching induced by centrifugal forces during molecular rotation, which can serve as a crucial parameter for evaluating the intermolecular interaction rigidity and the curvature of the PES in vdW complexes. Since *D* values are typically very small and highly sensitive to the higher-order derivatives of the PES, their accurate prediction has long been a major challenge in computational chemistry. Remarkably, the predicted *D* values in this work are in excellent agreement with the experimental data in both magnitude and isotopic evolutionary trends. A systematic comparison across the three complexes reveals a clear physical trend: as the atomic number and mass of the noble gas increase (Ar → Kr → Xe), the centrifugal distortion constant decreases systematically from ~0.9 kHz in Ar–CuF to ~0.2 kHz in Xe–CuF. This behavior is primarily attributed to the deeper potential wells and larger force constants inherent in the heavier noble gas systems. Consequently, these heavy complexes exhibit greater resistance to centrifugal stretching during rotation, which manifests spectroscopically as a systematic reduction in the *D* value.

### 2.5. Structural Parameters

An analysis of the bond lengths in the complexes provides a much deeper understanding of the nature of the intermolecular interactions and the effects of noble gas variation. Table 4 presents a detailed comparison of the structural parameters for the Rg–CuF complexes. Among these parameters, *r*_0_ represents the effective bond length directly fitted from rotational constants, *r*_1ε_ introduces a centrifugal distortion correction and *r*_m_^(1)^ further incorporates a mass-dependent correction to eliminate vibrational effects caused by isotopic substitution [8,9]. As shown in Table 4, compared to the experimental bond length of the free CuF monomer (1.7486 Å) [27], the uncorrected experimental Cu–F *r*_0_ values for Ar–CuF, Kr–CuF, and Xe–CuF exhibit a distinct systematic elongation. This elongation is not a genuine physical phenomenon but rather an artifact of vibrational averaging effects. Upon introducing centrifugal distortion and mass-dependent corrections to mitigate these vibrational contributions, the *r*_1ε_ value for Kr–CuF and the *r*_m_^(1)^ value for Xe–CuF contract to 1.7488 and 1.7498 Å, respectively, yielding negligible deviations of merely 0.0002 and 0.0011 Å from the free monomer bond length. This strongly indicates that the Cu–F fragment behaves as a nearly rigid structural unit within the complexes, regardless of the species or polarizability of the adjacent noble gas atom. This structural rigidity stands in stark contrast to that of the homologous Rg–AgF complexes. In the Ar–AgF, Kr–AgF, and Xe–AgF complexes, the Ag–F bond lengths are 1.98605 [3], 1.974567(68) [6], and 1.97099(12) Å [7], respectively. Compared to the free AgF monomer bond length (1.9832 Å) [18], the variations are +0.0029 Å, −0.0086 Å, and −0.0122 Å, respectively, demonstrating a significantly larger deformation magnitude than that observed in the Rg–CuF systems. Fundamentally, this discrepancy originates from their distinct electronic and structural properties: the Cu–F bond is inherently stronger and shorter than the Ag–F bond, rendering it naturally more resistant to perturbations from external atoms. Such rigidity not only reflects the higher effective nuclear charge of the copper atom but also corroborates the stronger covalent character of the Cu–F interaction. Furthermore, limited by the inherent precision of the structural parameters derived from experimental microwave spectroscopy, it is difficult to definitively determine from experimental data alone whether the Cu–F bond in the complexes undergoes a minute elongation or contraction relative to the free monomer. However, the theoretical Cu–F bond lengths calculated in this work perfectly reproduce the high-precision experimental values. This remarkable consistency not only validates the accuracy of the current computational methods but also justifies the theoretical modeling strategy of treating the CuF monomer as a nearly rigid rotor.

Notably, along the intermolecular interaction coordinate, the Rg–Cu bond lengths calculated in this work for the Kr–CuF and Xe–CuF complexes are 2.3192 Å and 2.4369 Å, respectively. The minimum deviations from the best matched experimental values are 0.0009 Å and 0.0042 Å, corresponding to relative errors of 0.03% and 0.17%, respectively. These errors are significantly larger than the computational deviations for the intramolecular Cu–F bond lengths (0.005% and 0.06%). This phenomenon indicates that the intermolecular interactions are the dominant factor contributing to the theoretical experimental discrepancies in the overall spectroscopic features. This is further corroborated by the error propagation relationship, which the magnitude of the relative errors for the calculated Rg–Cu bond lengths (0.03% and 0.17%) show a consistent trend with the percentage deviations obtained from the rotational constant calculations discussed earlier (0.003% and 0.07%).

### 2.6. Isotope Effects

**Figure 8:**
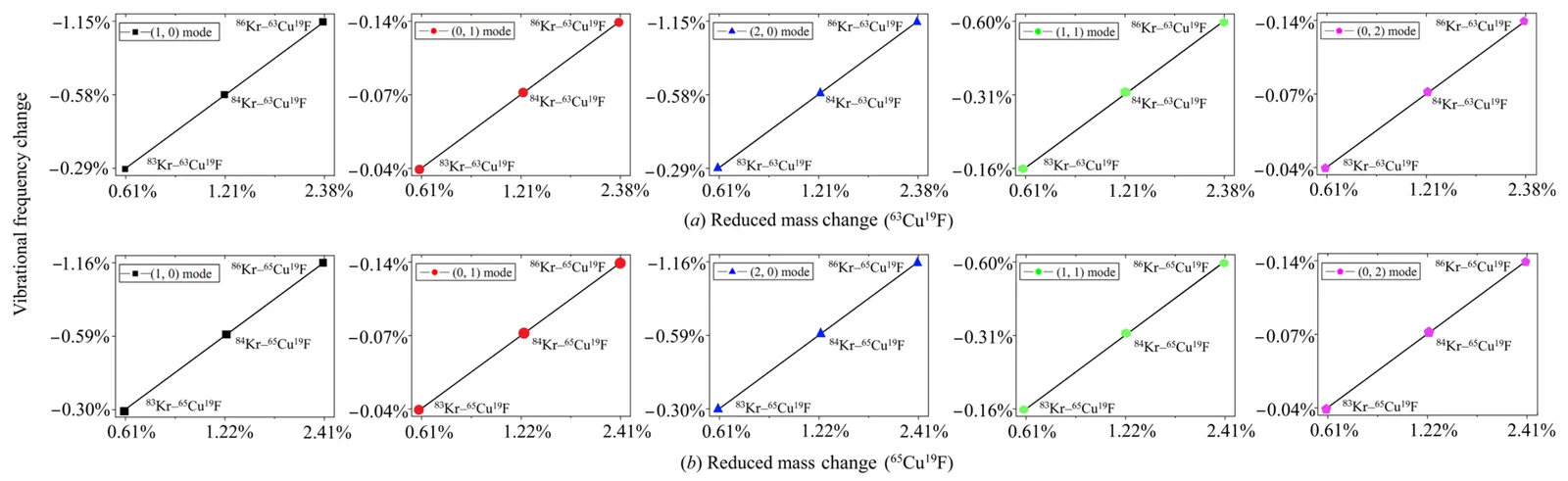
Plot of the change in vibrational frequency versus the change in reduced mass for the Kr–CuF isotopologues. (**a**) Reduced mass changes of ^63^Cu^19^F; (**b**) Reduced mass changes of ^65^Cu^19^F.

Isotopic substitution causes shifts in the spectroscopic parameters primarily through its effect on the reduced mass (*μ*). Figure 8 illustrates the correlation between the intermolecular vibrational frequencies and the reduced mass of the Kr–CuF isotopologues. Specifically, Figure 8a and Figure 8b present the data for the Kr-series isotopologues containing ^63^Cu and ^65^Cu, respectively. All five excited-state vibrational modes mentioned above, namely the fundamental stretching (1, 0) and bending (0, 1) modes, their overtones (2, 0) and (0, 2), and the combination band (1, 1), show a remarkable linear relationship. As the reduced mass increases, the changes in vibrational frequency exhibit a strictly monotonic decrease. This rigorous linear characteristic is highly consistent with the harmonic oscillator model. It not only provides compelling evidence that the intermolecular force constants remain invariant upon isotopic substitution but also further corroborates the negligible role of anharmonic effects in these low-lying excited vibrational states.

**Figure 9:**
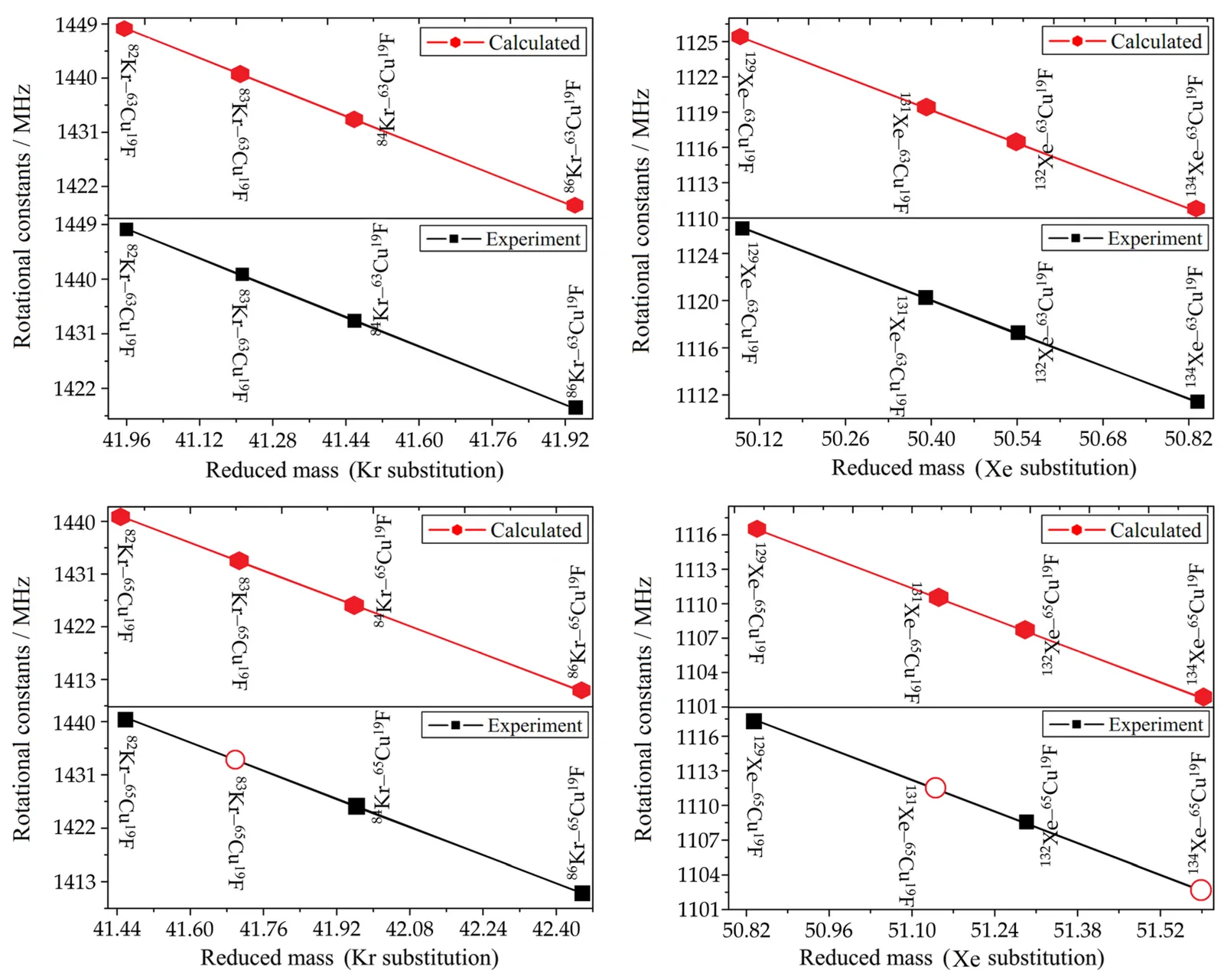
Plot of the rotational constant *B* versus the reduced mass for the isotopologues of the Kr–CuF and Xe–CuF complexes.

Figure 9 visually demonstrates the dependence of the rotational constants (*B*) on the reduced mass for the Kr–CuF and Xe–CuF systems. The four subplots correspond to the isotopic series of ^A^Kr–^63^Cu^19^F, ^A^Kr–^65^Cu^19^F, ^A^Xe–^63^Cu^19^F and ^A^Xe–^65^Cu^19^F, respectively. As depicted, both the calculated values (red ones) and the experimental observations (black ones) display a strictly linear decreasing trend with increasing reduced mass. More importantly, the slopes of the theoretical predictions are highly parallel to those of the experimental measurements, and their absolute values also in excellent agreement. According to the rigid-rotor model (*B* ∝ 1/*μR*^2^), the emergence of a strict linear relationship within a narrow range of isotopic mass variation strongly suggests that these noble-gas complexes possess extremely high structural rigidity. This result indicates that isotopic substitution does not induce any significant alteration in the molecular geometry, perfectly adhering to the premise of the Born-Oppenheimer approximation. Similar phenomena have also been reported in previous studies of systems such as Ar–CO_2_ [28], Ne–CO [25], and H_2_–CuF [29].

This precisely linear trend holds tremendous guiding value for spectroscopic experiments. Due to the relatively low natural abundances of certain isotopes and their extremely weak van der Waals binding nature, several isotopologues—such as ^83^Kr–^65^CuF, ^131^Xe–^65^CuF and ^134^Xe–^65^CuF—were not detected in FTMW experiments previously. By utilizing this linear relationship in conjunction with the highly accurate predictions derived from the CBS-level PES, the frequency search window for FTMW spectroscopy can be drastically narrowed from a broadband blind scan of several thousand MHz down to a targeted range of merely a few MHz, thereby enhancing the search efficiency. This predictive strategy was previously established and successfully validated by Wang et al. [18,19] in the Ar–AgX and Ar–CuX systems. By employing coordinate translation and PES re-interpolation, they accurately predicted the transition frequencies of various isotopologues, providing crucial theoretical navigation for the efficient assignment of subsequent experimental spectral lines.

### 2.7. Symmetry-Adapted Perturbation Theory (SAPT) Energy-Decomposition Analysis

To thoroughly characterize the physical nature of the Rg–Cu interaction, we performed SAPT calculations with Psi4 program [30] at the SAPT2+/aug-cc-pVQZ level. The aug-cc-pVQZ basis set was used for F and Ar. The ECP10MDF effective core potential was used for Cu and Kr, and the ECP28MDF potential was used for Xe. The calculations were performed at the CCSD(T)/aug-cc-pVQZ global-minimum geometries. These geometries are linear Rg–Cu–F with *θ* = 0° and *R* = 2.615, 2.713, and 2.838 Å for Ar, Kr, and Xe, respectively. To be consistent with the CCSD(T) calculations in Table 1, the SAPT calculations adopted the same frozen-monomer approximation, with the Cu–F bond length fixed at the experimental free-monomer value of 1.744923 Å [27]. The SAPT interaction energies were computed in the dimer-centered basis and are therefore free of basis set superposition error (BSSE).

SAPT2+ partitions the interaction energy into electrostatic (*E*_ele_), exchange (*E*_exc_), induction (*E*_ind_), and dispersion (*E*_dis_) contributions listed in Table 5. Following the standard SAPT2+ convention, the induction term includes the exchange–induction correction and the higher-order δ_HF_ correction. In Table 5, the induction energy is reported as a single total value, with the exchange–induction and δ_HF_ contributions term-by-term breakdown provided in Table S7 of the Supplementary Material. As shown in Table 5, the decomposition shows that induction is the dominant attractive term in all three complexes, contributing about 40% of the total attraction (39.6%, 39.1%, and 40.0% for Ar–CuF, Kr–CuF, and Xe–CuF), whereas electrostatics (27.2%, 31.1%, and 30.3%) and dispersion (33.2%, 29.8%, and 29.8%) play secondary roles. The substantial exchange–induction correction reflects pronounced orbital overlap and electron exchange between the monomers and is widely regarded as an energetic signature of charge-transfer or donor–acceptor character. These results demonstrate that the Rg–Cu interaction is not only a purely electrostatic or dispersive van der Waals interaction but also possesses substantial polarization and charge-transfer character with partial covalent nature.

We note that the SAPT2+ interaction energies are considerably smaller than the CCSD(T)/aug-cc-pVQZ binding energies. The total SAPT2+ interaction energies (−2067.6, −3280.4, and −3922.3 cm^−1^) amount to 54–69% of the CCSD(T)/aug-cc-pVQZ binding energies (−3813.8, −4776.0, and −6116.3 cm^−1^). This difference can be contributed to two main factors. First, the two methods differ in both the level of theory and the treatment of basis-set superposition error (BSSE). SAPT2+ is a perturbation expansion truncated at second order and augmented by the δ_HF_ correction. CCSD(T) is a nearly exact coupled-cluster method, and its binding energies were corrected for BSSE using the counterpoise method. For metal–noble-gas systems with dominant induction and large polarizability, low-order SAPT2+ perturbation theory systematically underestimates the binding. Second, strong charge transfer is reflected in large opposing terms. For Kr–CuF, the bare induction term $E_{ind}^{\left(20\right)}$ (−12,884.2 cm^−1^) and the exchange–induction term $E_{exch-ind}^{\left(20\right)}$ (+9278.9 cm^−1^) partially cancel, and the remaining induction components $E_{ind}^{\left(22\right)}$, $E_{exch-ind}^{\left(22\right)}$, and δ_HF_ bring the net induction to −2886.3 cm^−1^. Here, the individual terms are several times larger than their net sum because terms of opposite sign partially cancel. This cancellation pattern is the fingerprint of strong orbital overlap and charge transfer, and it also explains the slow convergence of low-order SAPT.

## 3. Computational Details

### 3.1. Ab Initio PESs Calculations

The intermolecular interaction of the Rg–CuF (Rg = Ar, Kr, Xe) complexes was described using Jacobi coordinates (*R*, *θ*). Herein, *R* denotes the distance from the Rg atom to the center of mass of the CuF monomer, and *θ* represents the angle between the *R* vector and the CuF molecular axis (oriented from Cu to F). The angle *θ* = 0*°* corresponds to the linear Rg–Cu–F global minimum, and *θ* = 180*°* to the anti-linear Rg–F–Cu local minimum. Within the rigid-rotor approximation, the CuF monomer was treated as a rigid rotor, with its intramolecular bond length fixed at the experimental value of *r*_e_ = 1.744923 Å [27].

All supermolecular single-point energies were calculated at the CCSD(T) level using Molpro 2010 [31]. The Dunning correlation-consistent basis sets, namely aug-cc-pVXZ (X = D, T, Q) [32], were employed for the F and Ar atoms. For the heavy atoms Cu, Kr, and Xe, effective core potential (ECP) basis sets that incorporate scalar relativistic effects were adopted: ECP10MDF [23] for Cu and Kr, and ECP28MDF [33] for Xe. To improve the description of intermolecular dispersion, a set of bond functions (3*s*3*p*2*d*1*f*1*g*) [34] was placed at the midpoint of the intermolecular distance *R* in all calculations, with exponents *α* = 0.9, 0.3, 0.1 for *s* and *p*; *α* = 0.6, 0.2 for *d*; *α* = 0.3 for *f* and *g*, respectively.

Basis set superposition error (BSSE) was corrected using the counterpoise procedure (CP) of Boys and Bernardi [24]. The interaction energy is given by:

(2) $$E_{\mathrm{int}}^{\mathrm{CP}}\left(R,\theta \right)=E_{\mathrm{complex}}\left(\Phi_{12};R,\theta \right)-E_{1}\left(\Phi_{12};R,\theta \right)-E_{2}\left(\Phi_{12};R,\theta \right)$$

where $E_{\mathrm{complex}}\left(\Phi_{12};R,\theta \right)$ is the total energy of the complex computed in the full basis set of monomers CuF and Rg. $E_{1}\left(\Phi_{12};R,\theta \right)$ and $E_{2}\left(\Phi_{12};R,\theta \right)$ denote the energies of CuF and Rg, respectively, calculated in the full basis set with the other monomer treated as a dummy atom.

### 3.2. PES Construction and CBS Extrapolation

The radial grids contained 24 points for Ar–CuF, 23 points for Kr–CuF, and 22 points for Xe–CuF, with the radial coordinate ranging from *R* = 1.50 to 10.00 Å. The angular grid covered *θ* = 0–180° in steps of 15°. The two-dimensional (2D) intermolecular PES *V*(*R*, *θ*) was obtained by one-dimensional Lagrange polynomial interpolation, applied first along the angular direction and then along the radial direction.

To approach the complete basis set (CBS) limit, we used a three-point extrapolation for the Hartree–Fock (HF) energy [35] and a two-point scheme for the correlation energy, and the extrapolation formulas are as follows:

(3) $$E_{HF}^{CBS}=\frac{E_{HF}^{X}E_{HF}^{X-2}-{\left(E_{HF}^{X-1}\right)}^{2}}{E_{HF}^{X}-2E_{HF}^{X-1}+E_{HF}^{X-2}}$$

(4) $$V_{\mathrm{CBS}}=\frac{X^{3}V_{X}-{\left(X-1\right)}^{3}V_{X-1}}{X^{3}-{\left(X-1\right)}^{3}}$$

In Equation (3), $E_{HF}^{CBS}$ is the HF energy extrapolated to the CBS limit $E_{HF}^{X}$, $E_{HF}^{X-1}$ and $E_{HF}^{X-2}$ represent the HF energies computed with three successive basis sets, respectively. In the present work, the cardinal numbers for the AVXZ (X = D, T, Q) basis sets are assigned as X = 4 for AVQZ, X = 3 for AVTZ, and X = 2 for AVDZ. Furthermore, in Equation (4), the correlation energy was extrapolated utilizing the *X*^3^ dependence [36].

### 3.3. Bound State Calculations

Within the rigid-rotor approximation, the Schrödinger equation for nuclear motion of the Rg–CuF complexes were solved in Jacobi coordinates. The Hamiltonian operator for the system can be written as [37]:

(5) $$\hat{H}=-\frac{{¯\;h}^{2}}{2\mu }\frac{\partial^{2}}{\partial R^{2}}+\frac{{¯\;h}^{2}}{2\mu R^{2}}{\left(\hat{J}-\hat{j}\right)}^{2}+B_{\mathrm{CuF}}{\hat{j}}^{2}+V\left(R,\theta \right)$$

where *μ* is the reduced mass of the complex, $\hat{J}$ and $\hat{j}$ are the total angular momentum and CuF monomer rotational angular momentum operators, and *B*_CuF_ is the rotational constant of the CuF monomer. The wave function was expanded as a linear combination of radial and angular basis functions:

(6) $$\psi \left(R,\theta \right)=\sum_{i}\sum_{j,K}c_{j,K}^{i}\varphi_{i}\left(R\right)\Theta_{j,K}^{JM\varepsilon }\left(\theta \right)$$

where $\varphi_{i}\left(R\right)$ is the radial sine basis function describing the intermolecular stretching vibration, and $\Theta_{j,K}^{JM\varepsilon }\left(\theta \right)$ is the angular basis function constructed from symmetry-adapted combinations of rotation matrix elements for the total angular momentum.

Eigenvalues and eigenfunctions were obtained by performing bound state calculation with the MPI-parallelized PARPACK package [38], via the implicitly restarted Arnoldi method. The calculations covered total angular momenta *J* = 0–6, with eigenvalue convergence accuracy better than 0.001 cm^−1^. The basis function parameters were set as follows: *N*_sine_ = 300 radial sine basis functions (integration range *R* = 3.0–16.0 bohr), angular basis truncation *j*_max_ = 300, and the potential energy matrix elements were evaluated using 310-point Gauss–Legendre numerical quadrature.

The intermolecular stretching (*n_s_*) and bending (*n_b_*) quantum numbers for each bound state were assigned by analyzing the probability distribution of the vibrational wave function in the (*R*, *θ*) space. The rotational constant *B* and centrifugal distortion constant *D* were determined by least-squares fitting of the bound state energies to an effective Hamiltonian using the SPFIT program [22], ensuring consistency with the treatment of FTMW experimental data.

Isotopic substitution effects were handled by the coordinate translation method [18,19]. Since the variation in isotopic mass leads to a slight shift in the center of mass of the system, the effective PES for each isotopologue was generated by coordinate translation and re-interpolation of the original PES. Bound state calculations on the PESs yielded the rovibrational levels and wavefunctions, which can be used to derive the vibrational frequencies, geometries, and spectroscopic parameters for all isotopologues of Ar, Kr, and Xe with natural abundances greater than 10% (spanning combinations of ^40^Ar, ^A^Kr (A = 82, 83, 84, 86), ^A^Xe (A = 129, 131, 132, 134) with ^A^Cu (A = 63, 65).

## 4. Conclusions

In this work, two-dimensional intermolecular potential energy surfaces were constructed for Rg–CuF (Rg = Ar, Kr, Xe) complexes at the CCSD(T) level, using augmented correlation-consistent basis sets aug-cc-pVXZ (X = D, T, Q) and complete basis set (CBS) extrapolation for the binding energies. The global minimum for each complex was identified as the linear Rg–Cu–F configuration, and the binding energy was found to increase substantially with the atomic number of the noble gas. Bound state calculations were performed on the optimal PESs to accurately predict the rotational constants and vibrational frequencies, with sub-MHz deviations from available experimental data. The localized nature of the vibrational wavefunctions demonstrates exceptional structural rigidity. Analysis of the isotope effects reveals a well-defined linear dependence of the rotational constant (*B*) on the reduced mass, a relationship that can be exploited for efficient prediction of unobserved isotopologues. Symmetry-adapted perturbation theory (SAPT) analysis further confirms that the Rg–Cu interaction has significant charge-transfer character and induction-dominated contributions, providing electronic-level evidence for the weak covalent nature of these bonds. Collectively, these findings indicate that the Rg–Cu bonds possess a weak covalent character, transcending the conventional van der Waals picture and offering new insights into noble gas chemistry.

## Figures and Tables

**Table 1:** Structural parameters and Energies of each geometry for the Rg–CuF (Rg = Ar, Kr, Xe) complexes at the CCSD(T) level (*R* in Å, *θ* in degree, and energy in cm^−1^).

Species	Methodology	Global Minimum	Local Minimum	Saddle Point
Ar–CuF	aug-cc-pVDZ	(2.649, 0.0, −3727.74)	(4.590, 180.0, −192.25)	(4.163, 135.0, −178.54)
	aug-cc-pVTZ	(2.628, 0.0, −3810.28)	(4.585, 180.0, −197.88)	(4.204, 139.0, −187.43)
	aug-cc-pVQZ	(2.621, 0.0, −3813.78)	(4.572, 180.0, −198.26)	(4.193, 139.0, −190.67)
	CBS-tz+qz	(2.615, 0.0, −3817.23)	(4.562, 180.0, −198.67)	(4.202, 140.0, −193.10)
Kr–CuF	aug-cc-pVDZ	(2.735, 0.0, −4786.84)	(4.694, 180.0, −243.91)	(4.279, 137.0, −228.28)
	aug-cc-pVTZ	(2.721, 0.0, −4814.82)	(4.683, 180.0, −254.78)	(4.300, 140.0, −241.88)
	aug-cc-pVQZ	(2.716, 0.0, −4775.99)	(4.670, 180.0, −259.89)	(4.286, 140.0, −248.85)
	CBS-dz+tz	(2.714, 0.0, −4828.53)	(4.678, 180.0, −259.44)	(4.303, 141.0, −247.88)
	CBS-tz+qz	(2.713, 0.0, −4748.32)	(4.661, 180.0, −263.76)	(4.293, 141.0, −254.09)
Xe–CuF	aug-cc-pVDZ	(2.861, 0.0, −6155.12)	(4.806, 180.0, −301.18)	(4.452, 140.0, −287.61)
	aug-cc-pVTZ	(2.848, 0.0, −6159.43)	(4.792, 180.0, −322.79)	(4.452, 142.0, −308.34)
	aug-cc-pVQZ	(2.842, 0.0, −6116.34)	(4.773, 180.0, −337.84)	(4.432, 142.0, −324.53)
	CBS-dz+tz	(2.843, 0.0, −6163.12)	(4.786, 180.0, −331.97)	(4.454, 143.0, −317.41)
	CBS-tz+qz	(2.838, 0.0, −6086.10)	(4.760, 180.0, −349.20)	(4.434, 143.0, −336.72)

**Table 2:** Comparison of rotational constants for the Rg–^63^Cu^19^F (Rg = ^40^Ar, ^82^Kr, ^132^Xe) isotopologues based on different basis set.

Species	Methodology	Rotational Constant/MHz	Δ*B/*MHz
^40^Ar–CuF	Experiment *^a^*	2197.036	0.000
	AVDZ	2157.410	39.626
	AVTZ	2185.176	11.86
	AVQZ	2195.199	1.837
	CBS	2196.004	1.032
^82^Kr–CuF	Experiment *^b^*	1448.247	0.000
	AVDZ	1434.901	13.346
	AVTZ	1448.207	0.040
	AVQZ	1452.105	−3.858
	CBS-dz+tz	1453.979	−5.732
	CBS-tz+qz	1454.965	−6.718
^132^Xe–CuF	Experiment *^c^*	1117.236	0.000
	AVDZ	1100.264	16.972
	AVTZ	1108.869	8.367
	AVQZ	1113.158	4.078
	CBS-dz+tz	1112.562	4.674
	CBS-tz+qz	1116.382	0.881

*^a^* Experimental data for the Ar–CuF was taken from Ref. [4]. *^b^* Experimental data for the Kr–CuF was taken from Ref. [8]. *^c^* Experimental data for the Xe–CuF was taken from Ref. [9].

**Table 3:** Predicted spectroscopic parameters for the Rg–CuF (Rg = Ar, Kr, Xe) complexes.

Species	Isotopologue	Experiment *^a^*		This work	
		*B/*MHz	*D/*kHz	*B/*MHz	*D/*kHz
Ar–CuF	Ar–^63^Cu^19^F	2197.0358	0.9416	2196.1027	0.8638
	Ar–^65^Cu^19^F	2193.1638	0.9427	2192.2355	0.9011
Kr–CuF	^82^Kr–^63^Cu^19^F	1448.2467	0.3862	1448.2072	0.3098
	^82^Kr–^65^Cu^19^F	1440.7921	0.3371	1440.7245	0.3147
	^83^Kr–^63^Cu^19^F	1440.6303	0.3890	1440.5987	0.4021
	^83^Kr–^65^Cu^19^F	–	–	1433.0489	0.3497
	^84^Kr–^63^Cu^19^F	1433.2064	0.3802	1433.1768	0.3147
	^84^Kr–^65^Cu^19^F	1425.6545	0.3727	1425.6038	0.3609
	^86^Kr–^63^Cu^19^F	1418.8143	0.3664	1418.7906	0.3584
	^86^Kr–^65^Cu^19^F	1411.1676	0.3636	1411.1242	0.3483
Xe–CuF	^129^Xe–^63^Cu^19^F	1126.1926	0.2040	1125.3341	0.1887
	^129^Xe–^65^Cu^19^F	1117.4820	0.2040	1116.6134	0.2096
	^131^Xe–^63^Cu^19^F	1120.1761	0.2070	1119.3245	0.2373
	^131^Xe–^65^Cu^19^F	–	–	1110.5550	0.1736
	^132^Xe–^63^Cu^19^F	1117.2361	0.1967	1116.3816	0.1916
	^132^Xe–^65^Cu^19^F	1108.4624	0.1930	1107.5970	0.1816
	^134^Xe–^63^Cu^19^F	1111.4750	0.1920	1110.6224	0.1729
	^134^Xe–^65^Cu^19^F	–	–	1101.7975	0.1771

*^a^* Experimental data for the Ar–CuF was taken from Ref. [4]; Experimental data for the Kr–CuF was taken from Ref. [8]; Experimental data for the Xe–CuF was taken from Ref. [9].

**Table 4:** Structural parameters of Rg–CuF (Rg = Ar, Kr, Xe) complexes.

Species	Methodology		*r*(Rg–Cu)/Å	*r*(Cu–F)/Å
Free monomer (CuF) *^a^*			–	1.7486
Ar–CuF	Experiment *^b^*	*r*_0_	2.2190	1.7530
	This work		2.2239	1.7488
Kr–CuF	Experiment *^c^*	*r*_0_	2.31801(61)	1.7536(13)
		*r*_1*ε*_	2.318301(40)	1.74883(17)
		*r*_m_^(1)^	2.316887(52)	1.74777(20)
	This work		2.3192	1.7479
Xe–CuF	Experiment *^d^*	*r*_0_	2.4327(5)	1.754(1)
		*r*_1*ε*_	2.43231(3)	1.7507(1)
		*r*_m_^(1)^	2.43099(5)	1.7498(1)
	This work		2.4369	1.7487

*^a^* Experimental data for the CuF was taken from Ref. [27]. *^b^* Experimental data for the Ar–CuF was taken from Ref. [4]. *^c^* Experimental data for the Kr–CuF was taken from Ref. [8]. *^d^* Experimental data for the Xe–CuF was taken from Ref. [9].

**Table 5:** SAPT2+/aug-cc-pVQZ energy decomposition of the Rg–CuF interaction at the linear (*θ* = 0°) global-minimum geometry of Table 1 (*R* = 2.615, 2.713, and 2.838 Å for Ar, Kr, and Xe, respectively). All energies are in cm^−1^.

Complex	*E*_ele_ *^a^*	*E*_exc_ *^b^*	*E*_ind_ *^c^*	*E*_dis_ *^d^*	*E*_SAPT_ *^e^*	*E*_CCSD(T)_ *^f^*	*E*_SAPT_/*E*_CCSD(T)_ *^g^*
Ar–CuF	−1242.6	2498.3	−1807.7	−1515.6	−2067.6	−3813.8	54%
Kr–CuF	−2297.0	4105.0	−2886.3	−2202.1	−3280.4	−4776.0	69%
Xe–CuF	−2277.9	3604.0	−3008.9	−2239.5	−3922.3	−6116.3	64%

*^a^ E*_ele_ is the total electrostatic interaction. *^b^ E*_exc_ is the total exchange (Pauli/steric) repulsion. *^c^ E*_ind_ is the total induction, including the δ_HF_ correction; see Table S7 for the term-by-term breakdown. *^d^ E*_dis_ is the total dispersion interaction. *^e^ E*_SAPT_ is the total SAPT interaction energy, *E*_SAPT_ = *E*_ele_ + *E*_exc_ + *E*_ind_ + *E*_dis_. *^f^* CCSD(T)/aug-cc-pVQZ binding energy from Table 1. *^g^* Ratio of the total SAPT energy to the CCSD(T) binding energy.

## Data Availability

The original contributions presented in this study are included in the article/Supplementary Materials. Further inquiries can be directed to the corresponding authors.

## Supplementary Materials

The following supporting information can be downloaded at https://www.mdpi.com/article/10.3390/molecules31173025/s1, Table S1: Calculated rotational energy levels (in cm^−1^) of Rg–^63^Cu^19^F (Rg = ^40^Ar, ^82^Kr, ^132^Xe) isotopologue for the basis set analysis; Table S2: Intermolecular vibrational modes (*n_s_*, *n_b_*) and vibrational frequencies (in cm^−1^) for the Kr–CuF and Xe–CuF complex; Table S3: Calculated rotational energy levels (in cm^−1^) of Rg–CuF complex; Table S4: Calculated rotational transition frequencies (in MHz) for the Ar–CuF complex; Table S5: Calculated rotational transition frequencies (in MHz) for the Kr–CuF complex; Table S6: Calculated rotational transition frequencies (in MHz) for the Xe–CuF complex; Table S7: Induction-term (*E*_ind_) breakdown of the SAPT2+/aug-cc-pVQZ decomposition (in cm^−1^).

## Abbreviations

The following abbreviations are used in this manuscript:

CCSD(T)	Coupled-cluster singles and doubles with non-iterative triples
CBS	Complete basis set
vdW	van der Waals
FTMW	Fourier transform microwave
NQCCs	Quadrupole coupling constants
PESs	Potential energy surfaces
SAPT	Symmetry-adapted perturbation theory
ECP	Effective core potential
BSSE	Basis set superposition error
HF	Hartree–Fock
