# Supplementary material for: Intermolecular Potential Energy Surfaces and Bound State Calculations of Rg–CuF (Rg = Ar, Kr, Xe): Insights into the Nature of Noble Gas–Metal Bonding
Source: Molecules. 2026 Aug 28;31(17):3025. doi: 10.3390/molecules31173025 (PMC13567284; doi:10.3390/molecules31173025)
Supplement: Supplementary file 1 [file molecules-31-03025-s001.zip › molecules-4516847-supplementary.pdf]

Table S1 Calculated rotational energy levels (in cm^{-1}) of Rg–$^{63}Cu^{19}F$ (Rg = ^{40}Ar, ^{82}Kr, ^{132}Xe) isotopologue for the basis set analysis.

Species	Methodology	$J = 0$	$J = 1$	$J = 2$	$J = 3$	$J = 4$	$J = 5$	$J = 6$
Ar–CuF	aug-cc-pVDZ	-3518.95748	-3518.81355	-3518.52570	-3518.09392	-3517.51822	-3516.79860	-3515.93506
	aug-cc-pVTZ	-3598.61458	-3598.46880	-3598.17724	-3597.73991	-3597.15680	-3596.42792	-3595.55327
	aug-cc-pVQZ	-3602.31790	-3602.17146	-3601.87856	-3601.43922	-3600.85344	-3600.12121	-3599.24255
	CBS	-3604.88169	-3604.73519	-3604.44218	-3604.00268	-3603.41668	-3602.68419	-3601.80521
Kr–CuF	aug-cc-pVDZ	-4596.01294	-4595.91721	-4595.72577	-4595.43860	-4595.05571	-4594.57710	-4594.00278
	aug-cc-pVTZ	-4621.87265	-4621.77604	-4621.58281	-4621.29297	-4620.90651	-4620.42345	-4619.84377
	aug-cc-pVQZ	-4584.12086	-4584.02399	-4583.83025	-4583.53963	-4583.15215	-4583.15215	-4582.08658
	CBS-DZ+TZ	-4635.30551	-4635.20851	-4635.01451	-4634.72352	-4634.33553	-4633.85054	-4633.26855
	CBS-TZ+QZ	-4557.02578	-4556.92871	-4556.73459	-4556.44339	-4556.05514	-4555.56982	-4554.98744
Xe–CuF	aug-cc-pVDZ	-5970.40704	-5970.33364	-5970.18683	-5969.96663	-5969.67302	-5969.30602	-5968.86561
	aug-cc-pVTZ	-5973.58295	-5973.50897	-5973.36102	-5973.13909	-5972.84319	-5972.47332	-5972.02947
	aug-cc-pVQZ	-5931.08419	-5931.00993	-5930.86141	-5930.63862	-5930.34158	-5929.97027	-5929.52470
	CBS-DZ+TZ	-5976.95669	-5976.88247	-5976.73402	-5976.51136	-5976.21447	-5975.84337	-5975.39804
	CBS-TZ+QZ	-5901.14392	-5901.06944	-5900.92049	-5900.69707	-5900.39917	-5900.02680	-5899.57995

Table S2 Intermolecular vibrational modes (n_s, n_b) and vibrational frequencies (in cm^{-1})for the Kr–CuF and Xe–CuF complex.

Kr–$^{63}Cu^{19}F$ isotopologues								
Mode	^{82}Kr–$^{63}Cu^{19}F$		^{83}Kr–$^{63}Cu^{19}F$		^{84}Kr–$^{63}Cu^{19}F$		^{86}Kr–$^{63}Cu^{19}F$	
(n_s, n_b)	Level	Frequency	Level	Frequency	Level	Frequency	Level	Frequency
(0, 0)	-4621.87	0.00	-4622.19	0.00	-4622.50	0.00	-4623.10	0.00
(1, 0)	-4441.70	180.17	-4442.55	179.64	-4443.38	179.12	-4444.99	178.10
(0, 1)	-4406.89	214.98	-4407.29	214.90	-4407.67	214.83	-4408.49	214.68
(2, 0)	-4264.95	356.93	-4266.31	355.88	-4267.64	354.86	-4270.22	352.87
(1, 1)	-4228.86	393.01	-4229.78	392.40	-4230.68	391.81	-4232.43	390.67
(0, 2)	-4194.79	427.09	-4195.28	426.93	-4195.72	426.78	-4196.61	426.49

Kr–^{65}Cu^{19}F isotopologues								
Mode	^{82}Kr–^{65}Cu^{19}F		^{83}Kr–^{65}Cu^{19}F		^{84}Kr–^{65}Cu^{19}F		^{86}Kr–^{65}Cu^{19}F	
(n_s, n_b)	Level	Frequency	Level	Frequency	Level	Frequency	Level	Frequency
(0, 0)	-4622.78	0.00	-4623.10	0.00	-4623.41	0.00	-4624.01	0.00
(1, 0)	-4443.66	179.12	-4444.52	178.58	-4445.35	178.06	-4446.97	177.04
(0, 1)	-4408.52	214.26	-4408.92	214.18	-4409.31	214.10	-4410.06	213.95
(2, 0)	-4267.92	354.86	-4269.29	353.81	-4270.62	352.78	-4273.22	350.79
(1, 1)	-4231.53	391.25	-4232.46	390.64	-4233.36	390.05	-4235.12	388.89
(0, 2)	-4197.11	425.67	-4197.59	425.51	-4198.05	425.36	-4198.95	425.06
Xe–^{63}Cu^{19}F isotopologues								
Mode	^{129}Xe–^{63}Cu^{19}F		^{131}Xe–^{63}Cu^{19}F		^{132}Xe–^{63}Cu^{19}F		^{134}Xe–^{63}Cu^{19}F	
(n_s, n_b)	Level	Frequency	Level	Frequency	Level	Frequency	Level	Frequency
(0, 0)	-5900.71	0.00	-5901.00	0.00	-5901.14	0.00	-5901.42	0.00
(1, 0)	-5727.59	173.12	-5728.39	172.61	-5728.78	172.37	-5729.54	171.88
(0, 1)	-5695.36	205.35	-5695.71	205.29	-5695.88	205.26	-5696.22	205.21
(2, 0)	-5556.93	343.78	-5558.22	342.78	-5558.85	342.29	-5560.09	341.34
(1, 1)	-5523.77	376.94	-5524.62	376.38	-5525.04	376.11	-5525.85	375.57
(0, 2)	-5492.05	408.66	-5492.46	408.54	-5492.66	408.49	-5493.05	408.37
Xe–^{65}Cu^{19}F isotopologues								
Mode	^{129}Xe–^{63}Cu^{19}F		^{131}Xe–^{63}Cu^{19}F		^{132}Xe–^{63}Cu^{19}F		^{134}Xe–^{63}Cu^{19}F	
(n_s, n_b)	Level	Frequency	Level	Frequency	Level	Frequency	Level	Frequency
(0, 0)	-5901.72	0.00	-5902.01	0.00	-5902.15	0.00	-5902.43	0.00
(1, 0)	-5729.84	171.88	-5730.64	171.37	-5731.03	171.12	-5731.80	170.63
(0, 1)	-5697.10	204.62	-5697.45	204.56	-5697.62	204.53	-5697.96	204.47
(2, 0)	-5560.39	341.33	-5561.69	340.32	-5562.32	339.83	-5563.57	338.86
(1, 1)	-5526.74	374.98	-5527.59	374.42	-5528.01	374.14	-5528.83	373.60
(0, 2)	-5494.50	407.22	-5494.91	407.10	-5495.11	407.04	-5495.50	406.93

Table S3 Calculated rotational energy levels (in cm^{-1}) of Rg–CuF complex.

Species	Isotopologues	$J = 0$	$J = 1$	$J = 2$	$J = 3$	$J = 4$	$J = 5$	$J = 6$
Ar–CuF	$Ar–^{63}Cu^{19}F$	-3604.87113	-3604.72462	-3604.43161	-3603.99209	-3603.40606	-3602.67353	-3601.79451
	$Ar–^{65}Cu^{19}F$	-3605.60049	-3605.45424	-3605.16174	-3604.72299	-3604.13800	-3603.40676	-3602.52929
Kr–CuF	$^{82}Kr–^{63}Cu^{19}F$	-4621.87265	-4621.77604	-4621.58281	-4621.29297	-4620.90651	-4620.42345	-4619.84377
	$^{82}Kr–^{65}Cu^{19}F$	-4622.78027	-4622.68416	-4622.49193	-4622.20358	-4621.81913	-4621.33856	-4620.76188
	$^{83}Kr–^{63}Cu^{19}F$	-4622.18850	-4622.09240	-4621.90018	-4621.61186	-4621.22744	-4620.74692	-4620.17029
	$^{83}Kr–^{65}Cu^{19}F$	-4623.09823	-4623.00263	-4622.81142	-4622.52461	-4622.14220	-4621.66419	-4621.09057
	$^{84}Kr–^{63}Cu^{19}F$	-4622.49665	-4622.40104	-4622.20981	-4621.92298	-4621.54054	-4621.06249	-4620.48883
	$^{84}Kr–^{65}Cu^{19}F$	-4623.40844	-4623.31334	-4623.12313	-4622.83781	-4622.45739	-4621.98187	-4621.41125
	$^{86}Kr–^{63}Cu^{19}F$	-4623.09508	-4623.00043	-4622.81113	-4622.52717	-4622.14857	-4621.67532	-4621.10742
	$^{86}Kr–^{65}Cu^{19}F$	-4624.01092	-4623.91678	-4623.72851	-4623.44609	-4623.06953	-4622.59884	-4622.03402
Xe–CuF	$^{129}Xe–^{63}Cu^{19}F$	-5900.70232	-5900.62725	-5900.47710	-5900.25188	-5899.95158	-5899.57621	-5899.12577
	$^{129}Xe–^{65}Cu^{19}F$	-5901.70646	-5901.63197	-5901.48298	-5901.25951	-5900.96154	-5900.58908	-5900.14213
	$^{131}Xe–^{63}Cu^{19}F$	-5900.99157	-5900.91690	-5900.76755	-5900.54353	-5900.24484	-5899.87148	-5899.42345
	$^{131}Xe–^{65}Cu^{19}F$	-5901.99796	-5901.92387	-5901.77569	-5901.55343	-5901.25708	-5900.88664	-5900.44211
	$^{132}Xe–^{63}Cu^{19}F$	-5901.13305	-5901.05857	-5900.90962	-5900.68619	-5900.38828	-5900.01590	-5899.56904
	$^{132}Xe–^{65}Cu^{19}F$	-5902.14054	-5902.06665	-5901.91886	-5901.69719	-5901.40163	-5901.03218	-5900.58884
	$^{134}Xe–^{63}Cu^{19}F$	-5901.41062	-5901.33653	-5901.18834	-5900.96607	-5900.66970	-5900.29924	-5899.85468
	$^{134}Xe–^{65}Cu^{19}F$	-5902.42028	-5902.34678	-5902.19977	-5901.97926	-5901.68524	-5901.31773	-5900.87671

Table S4 Calculated rotational transition frequencies (in MHz) for the Ar–CuF complex.

$J'\leftarrow J''$	Expt.		Ref.		CBS-TZ+QZ	
	Ar–^{63}Cu^{19}F	Ar–^{65}Cu^{19}F	Ar–^{63}Cu^{19}F	Ar–^{65}Cu^{19}F	Ar–^{63}Cu^{19}F	Ar–^{65}Cu^{19}F
1←0	4394.1	4386.3	4397.8 (-0.085%)	4390.1 (-0.085%)	4392.2 (0.043%)	4384.5 (0.042%)
2←1	8788.1	8772.6	8795.6 (-0.085%)	8780.1 (-0.085%)	8784.4 (0.042%)	8768.9 (0.042%)
3←2	13182.1	13158.9	13193.3 (-0.085%)	13170.1 (-0.085%)	13176.5 (0.042%)	13153.3 (0.042%)
4←3	17576.0	17545.1	17591.2 (-0.086%)	17560.0 (-0.085%)	17568.6 (0.042%)	17537.7 (0.042%)
5←4	21969.9	21931.2	21988.6 (-0.085%)	21949.9 (-0.085%)	21960.6 (0.042%)	21921.9 (0.042%)
6←5	26363.6	26317.2	26386.4 (-0.086%)	26339.5 (-0.085%)	26352.5 (0.042%)	26306.1 (0.042%)

Table S5 Calculated rotational transition frequencies (in MHz) for the Kr–CuF complex.

$J'\leftarrow J''$	^{82}Kr–^{63}Cu^{19}F		^{83}Kr–^{63}Cu^{19}F		^{84}Kr–^{63}Cu^{19}F		^{86}Kr–^{63}Cu^{19}F	
	Expt.	Calc.	Expt.	Calc.	Expt.	Calc.	Expt.	Calc.
1←0	2896.5	2896.3 (0.007%)	2881.3	2881.0(0.010%)	2866.4	2866.3 (0.003%)	2837.6	2837.5 (0.004%)
2←1	5793.0	5792.9 (0.002%)	5762.5	5762.6(-0.002%)	5732.8	5732.9 (-0.002%)	5675.2	5675.1 (0.002%)
3←2	8689.4	8689.2 (0.002%)	8643.7	8643.6(0.001%)	8599.2	8598.9 (0.003%)	8512.8	8512.9 (-0.001%)
4←3	11585.9	11585.8 (0.001%)	11524.9	11524.6(0.003%)	11465.6	11465.3 (0.003%)	11350.4	11350.1 (0.003%)
5←4	14482.3	14481.8 (0.003%)	14406.1	14405.6 (0.003%)	14331.9	14331.6 (0.002%)	14188.0	14187.7 (0.002%)
6←5	17378.6	17378.4 (0.001%)	17287.2	17286.9(0.002%)	17198.1	17197.9 (0.001%)	17025.5	17025.2 (0.002%)
$J'\leftarrow J''$	^{82}Kr–^{65}Cu^{19}F		^{83}Kr–^{65}Cu^{19}F		^{84}Kr–^{65}Cu^{19}F		^{86}Kr–^{65}Cu^{19}F	
	Expt.	Calc.	Expt.	Calc.	Expt.	Calc.	Expt.	Calc.
1←0	2881.6	2881.3 (0.010%)	—	2866.0	2851.3	2851.0 (0.011%)	2822.3	2822.2 (0.004%)
2←1	5763.2	5762.9 (0.005%)		5732.3	5702.6	5702.4 (0.004%)	5644.7	5644.2 (0.009%)
3←2	8644.7	8644.5 (0.002%)		8598.3	8553.9	8553.7 (0.002%)	8467.0	8466.7 (0.004%)
4←3	11526.3	11525.5 (0.007%)		11464.4	11405.1	11404.7 (0.004%)	11289.2	11289.0 (0.002%)
5←4	14407.8	14407.1 (0.005%)		14330.4	14256.4	14255.7 (0.005%)	14111.5	14110.9 (0.004%)
6←5	17289.2	17288.4 (0.005%)		17196.1	17107.5	17106.8 (0.004%)	16933.8	16933.1 (0.004%)

Table S6 Calculated rotational transition frequencies (in MHz) for the Xe–CuF complex.

J'←J''	^{129}Xe–^{63}Cu^{19}F		^{131}Xe–^{63}Cu^{19}F		^{132}Xe–^{63}Cu^{19}F		^{134}Xe–^{63}Cu^{19}F	
	Expt.	Calc.	Expt.	Calc.	Expt.	Calc.	Expt.	Calc.
1←0	2252.4	2250.7 (0.075%)	2240.4	2238.6 (0.080%)	2234.5	2234.5 (0.076%)	2222.9	2221.2 (0.076%)
2←1	4504.8	4501.3 (0.078%)	4480.7	4477.3 (0.076%)	4468.9	4468.9 (0.076%)	4445.9	4442.4 (0.079%)
3←2	6757.1	6752.0 (0.075%)	6721.0	6715.9 (0.076%)	6703.4	6703.4 (0.076%)	6668.8	6663.6 (0.078%)
4←3	9009.5	9002.7 (0.076%)	8961.4	8954.5 (0.076%)	8937.8	8937.8 (0.076%)	8891.8	8884.7 (0.080%)
5←4	11261.8	11253.2 (0.076%)	11201.7	11193.2 (0.076%)	11172.3	11172.3 (0.077%)	11114.7	11105.9 (0.079%)
6←5	13514.2	13503.9	13441.9	13431.7 (0.076%)	13406.7	13406.7 (0.077%)	13337.5	13327.0 (0.079%)
J'←J''	^{129}Xe–^{65}Cu^{19}F		^{131}Xe–^{65}Cu^{19}F		^{132}Xe–^{65}Cu^{19}F		^{134}Xe–^{65}Cu^{19}F	
	Expt.	Calc.	Expt.	Calc.	Expt.	Calc.	Expt.	Calc.
1←0	2235.0	2233.2 (0.081%)	—	2221.1	2216.9	2215.2 (0.077%)	—	2203.6
2←1	4469.9	4466.4 (0.078%)		4442.2	4433.8	4430.4 (0.077%)		4407.2
3←2	6704.9	6699.6 (0.079%)		6663.3	6650.8	6645.6 (0.078%)		6610.8
4←3	8939.8	8932.9 (0.077%)		8884.4	8867.6	8860.7 (0.078%)		8814.4
5←4	11174.7	11166.0 (0.078%)		11105.5	11084.5	11075.9 (0.078%)		11017.9
6←5	13409.6	13399.2 (0.078%)		13326.5	13301.4	13291.0 (0.078%)		13221.4

Table S7. Induction-term (E_{ind}) breakdown of the SAPT2+/aug-cc-pVQZ decomposition (in cm^{-1}).

Complex	$E_{ind}^{(20)}$ [a]	$E_{ind}^{(22)}$ [b]	$E_{exch-ind}^{(20)}$ [c]	$E_{exch-ind}^{(22)}$ [d]	δ_{HF} [e]	E_{ind} [f]
Ar–CuF	−5838.1	−1071.1	4118.4	755.6	227.5	−1807.7
Kr–CuF	−12884.2	−2122.7	9278.9	1528.7	1313.0	−2886.3
Xe–CuF	−14255.1	−1902.8	10055.7	1342.3	1751.0	−3008.9

[a] Second-order (uncoupled HF) induction: polarization of one monomer by the static field of the other. [b] Intramonomer correlation correction to the induction energy. [c] Exchange–induction correction: accounts for orbital overlap and electron exchange between the monomers; the indicator of charge-transfer (covalent) character. [d] Intramonomer correlation correction to the exchange–induction term. [e] δ_{HF}: infinite-order induction/charge-transfer correction evaluated at the HF level. [f] Total induction = $E_{ind}^{(20)} + E_{ind}^{(22)} + E_{exch-ind}^{(20)} + E_{exch-ind}^{(22)} + \delta_{HF}$.
